# The Centrosomal E3 Ubiquitin Ligase FBXO31-SCF Regulates Neuronal Morphogenesis and Migration

**DOI:** 10.1371/journal.pone.0057530

**Published:** 2013-02-28

**Authors:** Mayur Vadhvani, Nicola Schwedhelm-Domeyer, Chaitali Mukherjee, Judith Stegmüller

**Affiliations:** 1 Cellular and Molecular Neurobiology, Max Planck Institute of Experimental Medicine, Göttingen, Germany; 2 Center for Nanoscale Microscopy and Molecular Physiology of the Brain (CNMPB), Göttingen, Germany; Schepens Eye Research Institute, Harvard Medical School, United States of America

## Abstract

Neuronal development requires proper migration, polarization and establishment of axons and dendrites. Growing evidence identifies the ubiquitin proteasome system (UPS) with its numerous components as an important regulator of various aspects of neuronal development. F-box proteins are interchangeable subunits of the Cullin-1 based E3 ubiquitin ligase, but only a few family members have been studied. Here, we report that the centrosomal E3 ligase FBXO31-SCF (Skp1/Cullin-1/F-box protein) regulates neuronal morphogenesis and axonal identity. In addition, we identified the polarity protein Par6c as a novel interaction partner and substrate targeted for proteasomal degradation in the control of axon but not dendrite growth. Finally, we ascribe a role for FBXO31 in dendrite growth and neuronal migration in the developing cerebellar cortex. Taken together, we uncovered the centrosomal E3 ligase FBXO31-SCF as a novel regulator of neuronal development.

## Introduction

During brain development neurons acquire a typical polarized morphology, which is fundamental to proper functioning of the network. Both extrinsic as well as intrinsic programs contribute to neuronal morphogenesis. The ubiquitin proteasome system (UPS) has emerged as a crucial intrinsic regulator of neuronal morphogenesis and other aspects of neuronal development [1]–[3].

E3 ubiquitin ligases are the most numerous components of the UPS. They specifically recruit the substrate and the E2 ubiquitin-conjugating enzyme, which brings in the highly conserved small protein ubiquitin [4]. This interaction triggers the ubiquitination of the substrate and brings about degradation or functional modification of the target protein. Most E3 ligases belong to the RING (really interesting new gene)-type ligases, which share the E2-binding RING domain. RING ligases can either act as single molecule or as multi-subunit ligases [5]. The Cullin-1 based E3 ligase SCF (Skp1, Cullin-1, F-box protein) complex belongs to the latter; while the subunit Rbx1/Roc1 harbors the RING domain, the F-box protein represents an interchangeable subunit responsible for substrate recognition and recruitment. Interestingly, F-box proteins comprise a large family of approximately 70 members but only a few of them have been characterized in depth, mostly in the context of cell cycle regulation and thus cancer research [6]–[9]. F-box proteins have been classified into FBXW, FBXL and FBXO; while they share the F-box domain, the W-group harbors several WD40 repeats, the L-group leucine-rich repeats and the O-group other domains [6].

The quest for neuronal F-box proteins has only recently begun and revealed important functions for F-box proteins in the brain including stem cell differentiation, neuronal cell fate, cerebellar development, axon tract development, dendrite patterning, and synapse formation [10]–[16]. Here, we report that the centrosomal E3 ligase FBXO31-SCF controls neuronal morphogenesis and axonal identity. We identified the polarity protein Par6c as a novel substrate of FBXO31-SCF and established an FBXO31/Par6c pathway of axonal but not dendritic growth. In addition, we found that FBXO31 is required for dendrite growth and migration of neurons in the developing cerebellar cortex.

## Materials and Methods

### Ethics Statement

All experiments involving live animals have been conducted according to the animal protocol approved by the “Verbraucherschutz und Lebensmittelsicherheit” of Lower Saxony, Germany (33.11.42502-04-059/08).

### Plasmids and Antibodies

A DNA-based template method was used to express short hairpin RNAs. The sequences for shRNAs targeting FBXO31 are as follows: FBXO31 RNAi#1∶5′ GGATGAGTTCTCCACCAAGT 3′; FBXO31 RNAi#2∶5′ AGTCAGTACGACAACTGCCT 3′ and FBXO31 RNAi#3∶5′AGGGGCACCAAGATCACGGG 3′. The antibodies used for the study were the following: rb α-FBXO31 (Novus Biologicals), ms α-γ-tubulin (Sigma), ms α-AnkG (Neuromab), rb α-GFP (Invitrogen), ms α-myc (Santa Cruz), ms α-Flag (Santa Cruz), ms α-14-3-3ß (Santa Cruz), α-ubiquitin (DAKO), α-K48/K63 linkage-specific polyubiquitin (Millipore).

### Immunoprecipitation

293T cells, transfected with indicated plasmids, were lysed in Co-IP buffer (1% NP40, 150 mM NaCl, 20 mM Tris pH 7.4, 1 mM EDTA, 10% glycerol, protease inhibitors) and 5% of lysates were set aside as input. The remaining lysate (1 mg) was incubated with the primary antibody, followed by incubation with protein A sepharose. The precipitates were analyzed with SDS-PAGE followed by immunoblotting.

### Cell-based Ubiquitination Assay

Transfected HEK 293T cells were lysed in RIPA buffer (50 mM Tris-HCl pH 7.5, 150 mM NaCl, 1% NP40, 1% sodium deoxycholate and 5 mM EDTA) supplemented with fresh protease inhibitors (1 µg/mL pepstatin, 3 µg/mL aprotinin and 1 µg/mL leupeptin) and 10 mM N-ethylmaleimide (NEM). 1 mg of total protein was incubated with anti-myc antibody for immunoprecipitation, followed by incubation with protein A sepharose. The precipitates were washed twice with RIPA buffer, twice with lysis buffer (50 mM HEPES pH 7.5, 150 mM NaCl, 10% glycerol, 1.5 mM MgCl_2_, 1% Triton X-100) and boiled with SDS-sample buffer prior to immunoblotting analysis.

### Transfection of Primary Neurons

Cerebellar granule neurons were cultured and transfected 8 hours after plating using a modified calcium phosphate protocol as described earlier [33]. The calcium phosphate method ensures a 86%–95% co-transfection efficiency of multiple plasmids [34] To rule out the possibility that genetic manipulation (RNAi or overexpression) affects axon or dendrite length due to poor health, we co-transfected granule neurons in all experiment with a plasmid encoding BCL-X_L_ to stimulate neuronal survival. Expression of BCL-X_L_ does not affect neuronal morphology [33]. Neurons were fixed with paraformaldehyde after 3 to 4 days *in vitro* and subjected to immunocytochemistry.

### Centrosomal Purification

Transfected cerebellar granule neurons were treated with cytochalasin D (1 µg/mL) and nocodazole (0.2 µM) for 1 hour and lysed in lysis buffer (1 mM HEPES pH 7.2, 0.5% NP40, 0.5 mM MgCl_2_, 0.1% ß-mercaptoethanol) supplemented with protease inhibitors (1 µg/mL pepstatin, 1 µg/mL aprotinin, 1 µg/mL leupeptin and 1 mM PMSF). Cell lysates were subjected to gradient centrifugation and centrosomal fractions were purified using a discontinuous sucrose density gradient centrifugation (40%, 50% and 70% sucrose). The fractions were further analyzed using immunoblotting.

### In vivo Electroporation

P4 rat pups were electroporated as described previously (Konishi et al., 2004). Briefly, DNA together with Fast Green was injected into the cerebellar cortex of isofluorane-anesthetized P4 rat pups using a Hamilton syringe. Pups were subjected to 5 electrical pulses (160–170 V, 50 ms pulses, 950 ms pulse intervals) using a Harvard Apparatus ECM 830 square wave electroporator. Pups were sacrificed at P9 and 40 µm coronal sections of GFP-positive cerebella were analyzed.

### Morphometry

Images of transfected GFP-positive neurons were captured in a blinded manner using a Nikon Eclipse epifluorescence microscope. Axons and dendrites were manually traced to determine their lengths using ImageJ. For *in vivo* analysis, confocal z-stacks were processed in ImageJ and the cells were counted in individual layers and relative distance of migration was analyzed. Confocal images were captured using a Leica SP2 were processed using Imaris software (Bitplane) to determine the dendrite lengths of neurons in 3D.

## Results

### The Centrosomal E3 Ubiquitin Ligase FBXO31-SCF Regulates Neuronal Morphology

The E3 ligase FBXO31-SCF has been introduced as a tumor suppressor and cell cycle regulator [17], [18]. Interestingly, different sources suggest a brain-enriched expression of *FBXO31* [17] (CDT-DB at Riken). However FBXO31’s role in neurons remains elusive. To characterize the function of FBXO31, we started out by quantifying the expression of *FBXO31* in distinct brain regions including cortex, hippocampus, cerebellum and olfactory bulb using quantitative PCR and found a relative enrichment of *FBXO31* expression in postnatal and adult rat brain as compared to non-neural tissue (**Figure S1A–C, Methods S1**).

**Figure 1:**
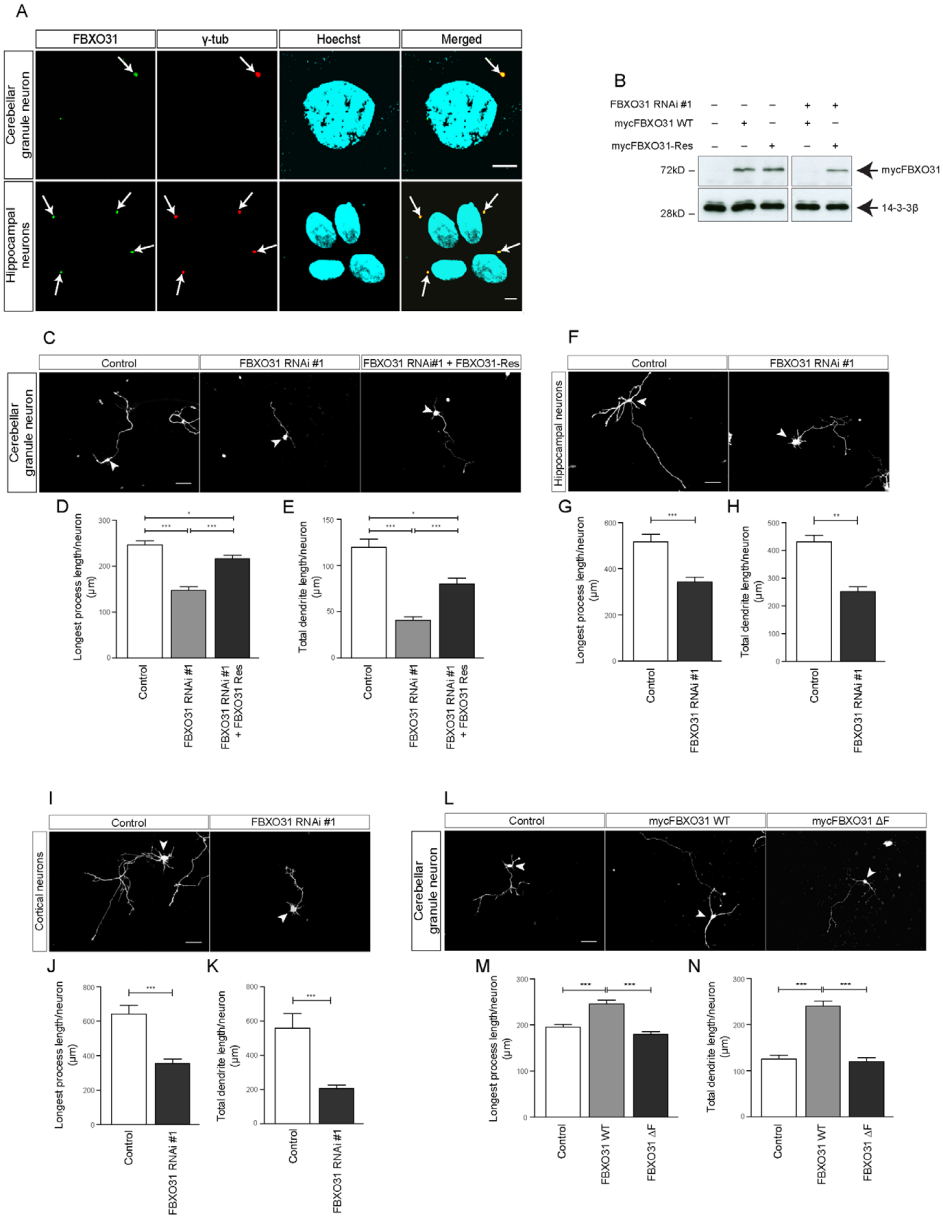
Centrosomal FBXO31 promotes axon and dendrite growth in neurons. A. Cultured cerebellar granule neurons and hippocampal neurons were fixed using methanol followed by immunostaining with α-FBXO31 and α-γtubulin antibodies. The cells were counterstained with the DNA dye bisbenzimide Hoechst 33258. Arrows indicate centrosomes. Scale bar equals 5 µm. **B.** Cell lysates of HEK 293T cells transfected with indicated plasmids were probed with α-myc antibody. 14-3- ß served as a loading control. **C.** Representative images of cerebellar granule neurons transfected with empty control vectors, FXO31 RNAi #1 plasmid or FBXO31 RNAi #1 together with mycFBXO31-Res at DIV 0 and analyzed at DIV 4. Arrowheads indicate granule neuron cell bodies. Scale bar equals 50 µm. **D.** Quantification of longest process lengths of granule neurons shown in C (N = 3, n = 296, mean±SEM, one-way ANOVA *p<0.05, ***p<0.001). **E.** Quantification of total dendrite lengths of granule neurons shown in C (N = 3, n = 291, mean±SEM, one-way ANOVA, *p<0.05, ***p<0.001). **F.** Representative images of cultured hippocampal neurons transfected with control vector or FBXO31 RNAi #1 plasmids at DIV 1 and analyzed at DIV 5. Arrowheads indicate hippocampal neuron cell bodies. Scale bar equals 50 µm. **G.** Quantification of longest process lengths of hippocampal neurons shown in F (N = 3, n = 190, mean±SEM, unpaired t-test, ***p<0.001). **H.** Quantification of total dendrite lengths of hippocampal neurons shown in F (N = 3, n = 184, mean±SEM, unpaired t-test, **p<0.01). **I.** Representative images of cultured cortical neurons transfected with control vector or FBXO31 RNAi #1 plasmids at DIV 1 and analyzed at DIV 5. Arrowheads indicate cortical neuron cell bodies. Scale bar equals 50 µm. **J.** Quantification of longest process lengths of cortical neurons shown in I (N = 3, n = 164, mean±SEM, unpaired t-test, ***p<0.001). **K.** Quantification of total dendrite lengths of cortical neurons shown in I (N = 3, n = 147, mean±SEM, unpaired t-test, ***p<0.001). **L.** Representative images of cerebellar granule neurons transfected with empty control vector, mycFBXO31 wild type (WT) plasmid or mycFBXO31 ΔF mutant plasmid at DIV 0 and analyzed at DIV 3. Arrowheads indicate granule neuron cell bodies. Scale bar equals 50 µm. **M.** Quantification of longest process lengths of granule neurons shown in L (N = 3, n = 381, mean±SEM, one-way ANOVA ***p<0.001). **N.** Quantification of total dendrite lengths of granule neurons shown in L (N = 3, n = 341, mean±SEM, one-way ANOVA, ***p<0.001).

We then determined the localization of FBXO31 in neurons and discovered that FBXO31 localizes to the centrosome in both cerebellar granule neurons and in hippocampal neurons (**Figure 1A**). Co-staining with the centrosomal protein -tubulin supported FBXO31’s centrosomal localization (**Figure 1A**). In addition, we confirmed the specificity of the immunostaining in neurons by preincubation of the antibody with recombinant FBXO31 protein (**Figure S1D)**. Interestingly, a previous study described the SCF complex subunit Skp1 and Cullin-1 as components of the centrosomal material [19], which prompted us to determine if the binding to Skp1 or Cullin-1 is required to recruit FBXO31 to the centrosome. An FBXO31 mutant lacking the F-box domain, which mediates the binding to Skp1 and Cullin-1 (**Figure S2**) [17], [20], still localizes to the centrosome. Further mapping analysis revealed that a stretch in the N-terminal region of FBXO31 is required for its recruitment to the centrosome (**Figure S3**).

As a component of the centrosomal material, we reasoned that FBXO31 might play a role in neuronal morphogenesis or migration. We used an RNAi approach to acutely knockdown FBXO31 in neurons. Validation of FBXO31 RNAi plasmids in heterologous cells demonstrated that two of the RNAi plasmids efficiently downregulate FBXO31, while one had no effect and served as a negative control thereafter (**Figure S4A).** We then transfected neurons with the control vector or different FBXO31 RNAi plasmids together with the GFP expression plasmid. We found that while the non-functional RNAi plasmid has little or no effect on axon (longest process) and dendrite growth, the functional RNAi plasmids lead to a decrease in both axonal and dendritic length (**Figure 1C–E**, **Figure S4B–D)**. To ensure that the morphological FBXO31 RNAi phenotype is specific and to rule out off-target effects, we constructed an RNAi-resistant form of FBXO31 (FBXO31-Rescue = FBXO31-Res). We introduced silent mutations into the RNAi target region and validated the FBXO31-Res encoding plasmid in heterologous cells **(** **Figure 1B**). We observed that while FBXO31 RNAi decreases axon (longest process) and dendrite length, expression of FBXO31-Res leads to significantly longer axons and dendrites as compared to FBXO31 knockdown neurons, supporting the specific FBXO31 RNAi-induced phenotype (**Figure 1C–E** **)**. In addition, we analyzed whether FBXO31 knockdown affects neuronal survival. Here, we transfected neurons with control vector or FBXO31 RNAi plasmids together with the ß-Galactosidase expression plasmid and assessed neuronal survival. We found a slight increase in apoptosis in FBXO31 knockdown neurons as compared to control (**Figure S4E**) and concluded that FBXO31 plays a rather subordinate role in neuronal survival. To determine if FBXO31-mediated neuronal morphogenesis is a generalizable mechanism, we examined FBXO31 knockdown in hippocampal and in cortical neurons and found that FBXO31 promotes axon (longest process) and dendrite growth in both neuronal cell types (**Figure 1F–K**). These data indicate that FBXO31 promotes axon and dendrite growth.

In gain-of function analyses, we examined the effect of overexpression of FBXO31. Conversely to the loss-of-function phenotype, we found that FBXO31 overexpression results in longer axons and dendrites, underscoring the role of FBXO31 in neuronal morphogenesis (**Figure 1L–N**). Furthermore, we found that ligase activity of FBXO31-SCF is required to promote neuronal morphogenesis, since the aforementioned FBXO31 ΔF-box mutant fails to promote elongation of the processes (**Figure 1L–N**). These data bolster our finding that FBXO31 acts as a regulator of neuronal morphogenesis.

### FBXO31-SCF Controls Axonal Identity

**Figure 2:**
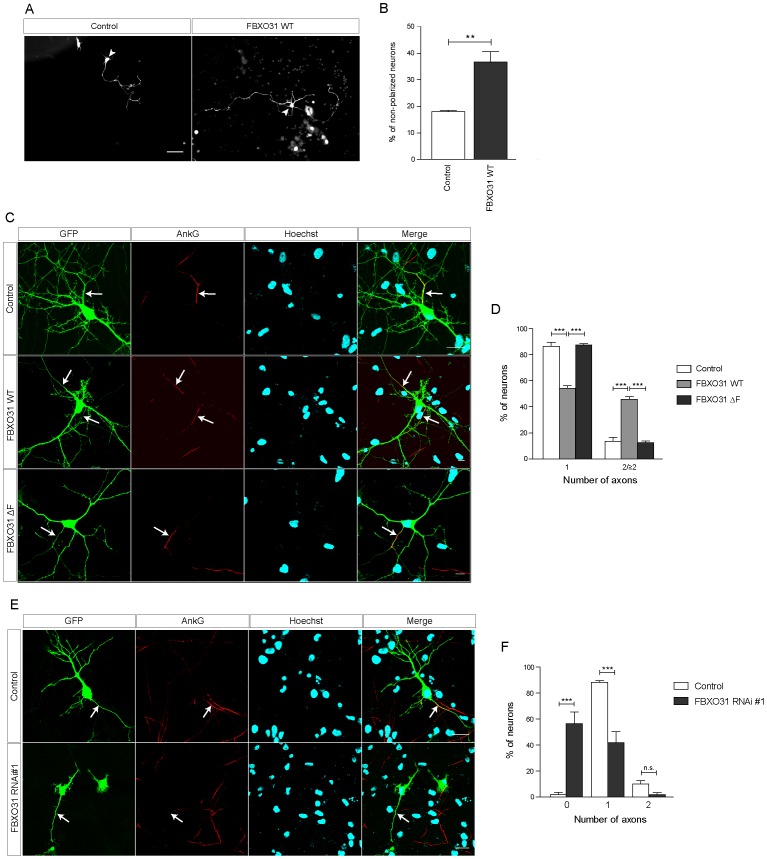
FBXO31 regulates axonal identity in neurons. A. Representative images of cerebellar granule neurons transfected with control vector or mycFBXO31 WT plasmid at DIV 0 and analyzed at DIV 3. Arrowheads indicate granule neuron cell bodies. Scale bar equals 50 µm. **B.** Quantification of percentage of non-polarized granule neurons shown in A. (N = 3, n = 256, mean±SEM, unpaired t-test, **p<0.01). **C.** Representative images of cultured hippocampal neurons transfected at DIV 1 with control vector, plasmids encoding mycFBXO31 WT or mycFBXO31 ΔF together with the GFP plasmid and immunostained at DIV 7 with α-GFP and α-AnkG antibodies and counterstained with Hoechst. Arrows indicate axon initial segment. Scale bar equals 10 µm. **D.** Quantification of number of axons in C. A total of 169 cells were analyzed (N = 3, mean±SEM, two-way ANOVA ***p<0.001). **E.** Representative images of cultured hippocampal neurons from E18 rat embryos transfected with control vector or FBXO31 RNAi#1/CMVGFP plasmid at DIV 1 and immunostained at DIV 6 with α-GFP and α-AnkG antibody and counterstained with Hoechst. Arrows indicate axon initial segment. Scale bar equals 10 µm. **F.** Quantification of number of axons in E. A total of 121 cells were analyzed (N = 3, mean±SEM, two-way ANOVA ***p<0.001).

Aside from the enhanced of neurite growth, we noticed that a larger number of FBXO31-overexpressing granule neurons appeared non-polarized as compared to control neurons. We carried out morphological analysis and defined a neuron as polarized when the longest process was at least twice as long as the second longest. When we quantified the number of non-polarized neurons, we found that while 18% of control neurons appear non-polarized, 38% of FBXO31-overexpressing neurons display a non-polarized morphology (**Figure 2A, 2B**). We then used molecular markers to examine a possible defect. Axons harbor several unique features including the axon initial segment (AIS), which is characterized by the presence of AnkyrinG. AnkyrinG is responsible for the organization of the AIS and the maintenance of neuronal polarity [21], [22]. We subjected control vector-, wild type FBXO31- and FBXO31 ΔF-expressing hippocampal neurons to immunocytochemistry and found that a significantly larger number of wild type FBXO31-expressing neurons harbor two or more AnkG-positive processes while control or FBXO31 ΔF neurons display mostly one AnkG-positive axon (**Figure 2C, 2D**). Conversely, when we triggered FBXO31 knockdown, a large percentage of longest processes failed to display the axonal marker AnkyrinG (**Figure 2E, 2F**). These data suggest that FBXO31 controls not only neuronal morphogenesis but also axonal identity.

### Par6c is a Novel Target of FBXO31-SCF in the Control of Axon Growth

**Figure 3:**
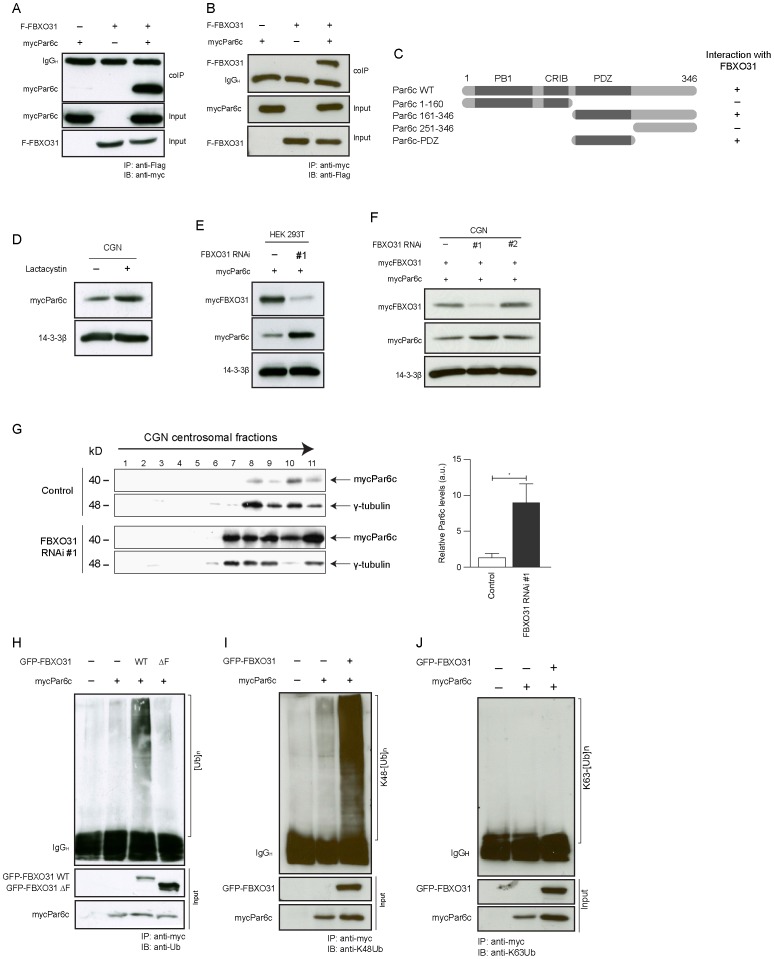
FBXO31-SCF targets Par6c to proteasomal degradation. A. Transfected HEK 293T cells lysates were subjected to immunoprecipitation with α-Flag antibody and immunoblotted with α-myc antibody. **B.** Transfected HEK 293T cells lysates were subjected to immunoprecipitation with α-myc antibody and immunoblotted with α-Flag antibody. **C.** Schematic showing various Par6c deletion mutants and their interaction with FBXO31. **D.** Granule neurons were transfected with Par6c plasmid at DIV0 and treated with DMSO or lactacystin for 10 hours prior to lysis at DIV 3. Lysates were immunoblotted with α-myc antibody. 14-3-3ß served as a loading control. **E.** and **F.** HEK 293T cells (E) and granule neurons (F) were transfected with mycPar6c plasmid together with FBXO31 RNAi plasmids or respective control vectors as indicated. Cell lysates were immunoblotted with α-myc and α-Flag antibodies. 14-3-3ß served as a loading control. **G.** Cerebellar granule neurons were transfected with mycPar6c plasmid together with FBXO31 RNAi #1 plasmid or respective control vector at DIV 2. Centrosomal purification was performed at DIV 6 using sucrose density gradient centrifugation. The fractions were probed with α-myc antibody. γ-tubulin served as a positive control for centrosomal protein. The histogram shows Par6c levels relative to γ-tubulin at the centrosome (N = 3, mean±SEM, unpaired t-test, *p<0.05). **H.** HEK 293T cells were co-transfected with mycPar6c and GFP-FBXO31 WT or ΔF plasmids together with respective control vectors. Cell lysates were subjected to immunoprecipitation with α-myc antibody and immunoblotted with α-ubiquitin antibody. **I.** HEK 293T cells were co-transfected with mycPar6c and GFP-FBXO31 WT plasmids together with respective control vectors. Cell lysates were subjected to immunoprecipitation with α-myc antibody and immunoblotted with K48-specific α-ubiquitin antibody. **J.** HEK 293T cells were co-transfected with mycPar6c and GFP-FBXO31 WT plasmids together with respective control vectors. Cell lysates were subjected to immunoprecipitation with anti-myc antibody and immunoblotted with K63-specific anti-ubiquitin antibody.

To identify targets of FBXO31-SCF and owing to FBXO31’s centrosomal localization, we took a candidate approach and found that FBXO31 interacts with Par6c, a previously identified centrosomal protein [23], [24]. Par6 is known to regulate epithelial cell polarity [25], [26] and neuronal polarity [27], [28]. In the nervous system, Par6c represents the predominantly enriched Par6 family member [29]. We determined the interaction of exogenous FBXO31 and Par6c in heterologous cells by immunoprecipitating FBXO31 followed by immunoblotting for Par6c (**Figure 3A**). In a reciprocal experiment, we immunoprecipitated Par6c and immunoblotted for FBXO31 (**Figure 3B**). Mapping analyses of FBXO31 and Par6c revealed that Par6c’s PDZ domain mediates the interaction with FBXO31 (**Figure 3C** **, Figure S5A**). These experiments establish the FBXO31-Par6c interaction.

To determine if Par6c is a potential substrate of FBXO31-SCF, we first examined if Par6c levels respond to proteasome inhibition. We transfected neurons with the Par6c expression plasmid and subjected them to treatment with the proteasome inhibitor lactacystin or vehicle as control. We analyzed the lysates and found that proteasome inhibition results in the accumulation of Par6c (**Figure 3D**). In further experiments, we analyzed if Par6c levels respond to presence or absence of FBXO31. We expressed the Par6c plasmid together with control vector or the FBXO31 plasmid in heterologous cells and found that overexpression of FBXO31 results in decreased Par6c levels while the control protein 14-3-3ß remains unaffected (**Figure S5B**). Conversely, the expression of the Par6c and FBXO31 plasmids together with control vector or FBXO31 RNAi plasmid results in an accumulation of Par6c in response to low FBXO31 levels but no change in 14-3-3ß (**Figure 3E**). To corroborate this finding in neurons, we compared Par6c levels in neurons upon FBXO1 overexpression and knockdown, respectively. Immunoblotting of neuronal lysates revealed that Par6c levels are reduced when FBXO31 is overexpressed (**Figure S5C**) and increased upon FBXO31 RNAi (**Figure 3F**). For the latter we included a non-functional FBXO31 RNAi plasmid as control and observed no change of Par6c levels (**Figure 3F**). In further experiments, we carried out centrosomal purification analysis to determine if centrosomal Par6c is affected by FBXO31 knockdown. We induced FBXO31 RNAi in neurons and subjected the lysate to an ultracentrifugation procedure to enrich for centrosomes. In control conditions, we find that Par6c appears together with the centrosomal protein gαμμα−tubulin in the bottom fractions. It is also here where we observe the accumulation of Par6c upon FBXO31 knockdown (**Figure 3G**). We do not detect any Par6c in the supernatant, where non-centrosomal proteins including 14-3-3ß are present (**Figure S5D**). This could mean that cytoplasmic Parc6c is below detection-level or that Par6c is solely present at the centrosome at the time when we examine the neurons. Collectively, these data suggest that centrosomal Par6c is targeted for degradation by FBXO31.

To examine if FBXO31-SCF is the ligase responsible for Par6c ubiquitination, we expressed Par6c together with control vector, a plasmid expressing wild type FBXO31 or the ligase-dead mutant FBXO31 ΔF-box. The lysates were subjected to immunoprecipitation for Par6c and immunoblotted with the ubiquitin antibody. While we found Par6c to be sparsely ubiquitinated in control or FBXO31-ΔF conditions, wild type FBXO31 potently stimulates polyubiquitination of Par6c (**Figure 3H**). To confirm that the polyubiquitination of Par6c contributes to its proteasomal turnover, we examined the linkage of the polyubiquitination chain associated with Par6c. Ubiquitin chains can be assembled via different lysines in ubiquitin. Ubiquitin chains that are linked via lysine 48 (K48) are known to trigger the degradation of proteins, while K63-linkage of ubiquitin represents a non-proteolytic modification [30], [31]. In cell-based ubiquitination assays, we found that FBXO31 triggers the assembly of a K48-linked but not K63-linked polyubiquitin chain of Par6c (**Figure 3I, 3J**). In addition, we carried out this cell based-ubiquitination assay using denatured lysates to exclude that Par6c interactors are being detected by ubiquitin. As demonstrated before, we found that Par6c is modified by K48-linked ubiquitin chains only in the presence of wild type but not ligase-dead FBXO31 (**Figure S5E,** **Methods S2**). Collectively, our data indicate that Par6c is targeted for proteasomal degradation by FBXO31-SCF.

### Par6c Suppresses Axon Growth

**Figure 4:**
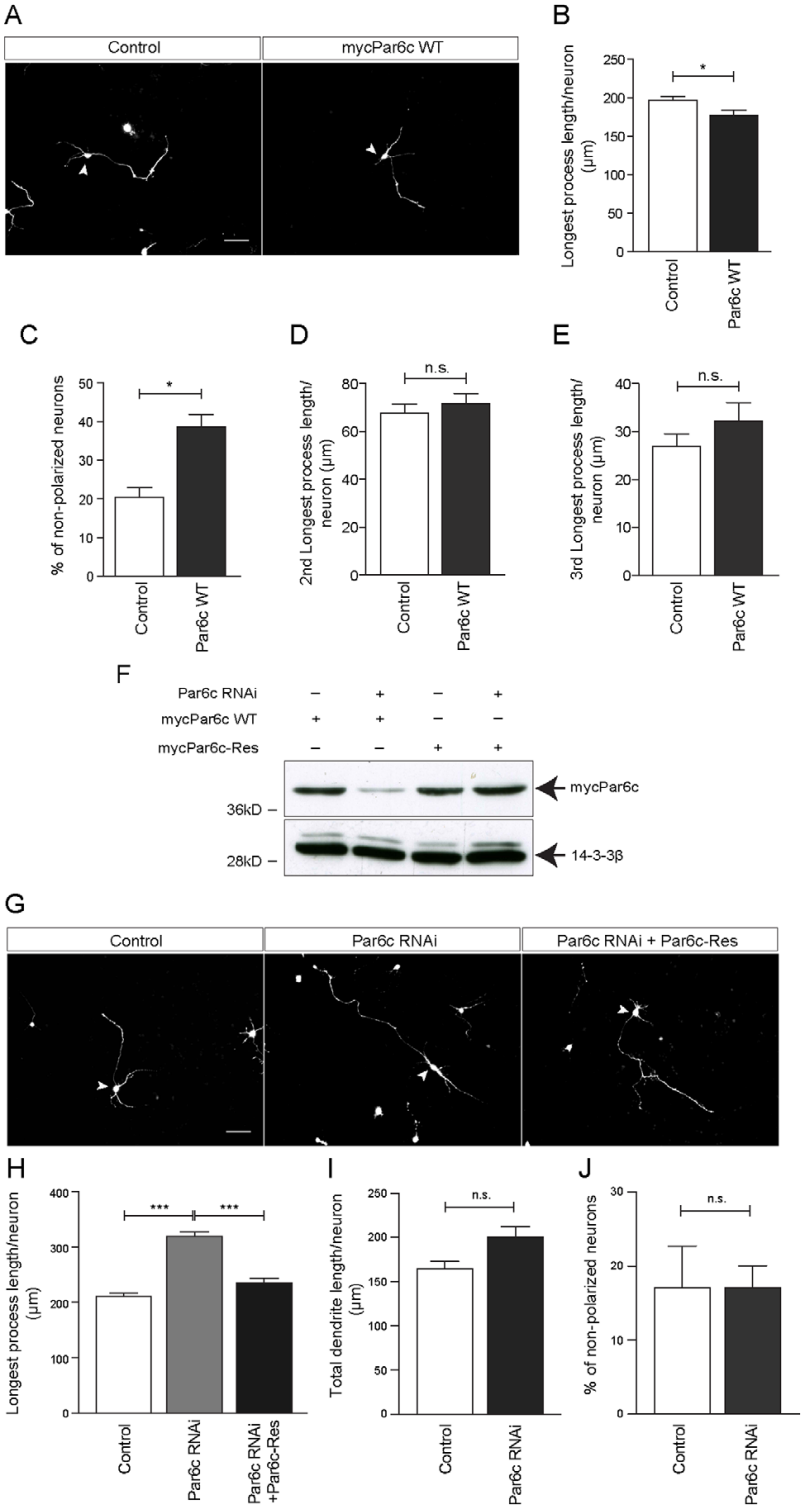
Par6c acts as an axon growth suppressor. A. Representative images of granule neurons transfected with control vector or plasmid encoding mycPar6c WT together with the GFP plasmid at DIV 0 and analyzed at DIV 3. Arrowheads indicate granule neurons cell bodies. Scale bar represents 50 µm. **B.** Quantification of longest process length of granule neurons shown in A. (N = 3, n = 160, mean±SEM, unpaired t-test, *p<0.05). **C.** Quantification of percentage of non-polarized granule neurons shown in A. (N = 3, n = 226, mean±SEM, unpaired t-test, *p<0.05). **D. and E.** Quantification of 2^nd^ longest (D) and 3^rd^ longest process length (E) of granule neurons shown in A. (N = 3, n = 160, mean±SEM, unpaired t-test, *p<0.05). **F.** HEK 293T cell lysates transfected with mycPar6c WT or mycPar6c-Res plasmids together with control or Par6c RNAi plasmids were immunoblotted with α-myc antibody. 14-3-3ß served as a loading control. **G.** Representative images of granule neurons transfected with control vector or Par6c RNAi or Par6c RNAi and Par6c-Res together with the GFP plasmid at DIV 0 and analyzed at DIV 4. Arrowheads indicate granule neuron cell bodies. Scale bar equals 50 µm. **H.** Quantification of longest process length of granule neurons transfected with control vector or Par6c RNAi plasmid or both Par6c RNAi plasmid and mycPar6c-Res plasmid together with GFP plasmid at DIV 0 and analyzed at DIV 4 (N = 3, n = 309, mean±SEM, one-way ANOVA, ***p<0.001) **I.** Quantification of total dendrite lengths of granule neurons transfected with control vector or Par6c RNAi plasmid together with GFP plasmid at DIV 0 and analyzed at DIV 4 (N = 3, n = 255, mean±SEM, one-way ANOVA, n.s. = not significant). **J.** Quantification of percentage of non-polarized granule neurons transfected with control vector or Par6c RNAi plasmid together with GFP plasmid at DIV 0 and analyzed at DIV 4. (N = 3, n = 313, mean±SEM, one-way ANOVA, n.s. = not significant).

We then examined if Par6c has in addition to its role in polarity, neurite growth-regulating functions. Since Par6c levels are regulated by FBXO31-SCF, we reasoned that overexpression of Par6c may phenocopy the FBXO31 RNAi phenotype. To acknowledge that both FBXO31 and Par6c affect axonal identity and polarity, we refer to the axon as longest process. Indeed, we found that Par6c gain-of-function results in reduced longest process growth and thus a shift towards a larger number of non-polarized neurons (**Figure 4A–C**). The lengths of the second and third longest processes however are left unaffected by Par6c overexpression. (**Figure 4D, 4E**). To investigate a possible role of the only other known substrate of FBXO31 in neuronal morphogenesis [18], we tested if Cyclin D1 overexpression has any effects on process length or polarity. However, we found that neither is the case (**Figure S6A–D**) Subsequently, we investigated loss-of-function of Par6c in neurons and generated a Par6c RNAi plasmid using the targeting region described by Zhang and Macara [32]. The Par6c RNAi plasmid was validated in heterologous cells using immunoblotting (**Figure 4F**). We then transfected neurons with control vector or Par6c RNAi plasmid and found that Par6c knockdown enhances longest process growth but has neither an effect on dendrites nor on the number of non-polarized neurons (**Figure 4G–J**). To ensure the specificity of the Par6c knockdown phenotype, we generated a Par6c rescue expression plasmid ( = Par6c-Res) by introducing silent mutations in the targeting region that renders Par6c insensitive to RNAi. The sustained expression of Par6c-Res under Par6c knockdown was confirmed in heterologous cells (**Figure 4F**). We then transfected neurons with either control vectors, the Par6c RNAi plasmid and control vector or the Par6c RNAi plasmid together with the Par6c-Res plasmid and found that the latter rescues the Par6c RNAi phenotype and reduces longest process length to baseline levels of control neurons (**Figure 4G, 4H**), but has no significant effects on dendrite length or polarity (**Figure 4I, 4J**). These experiments indicate that Par6c acts as a suppressor of longest process growth.

### An FBXO31-SCF/Par6c Pathway of Neuronal Morphogenesis

**Figure 5:**
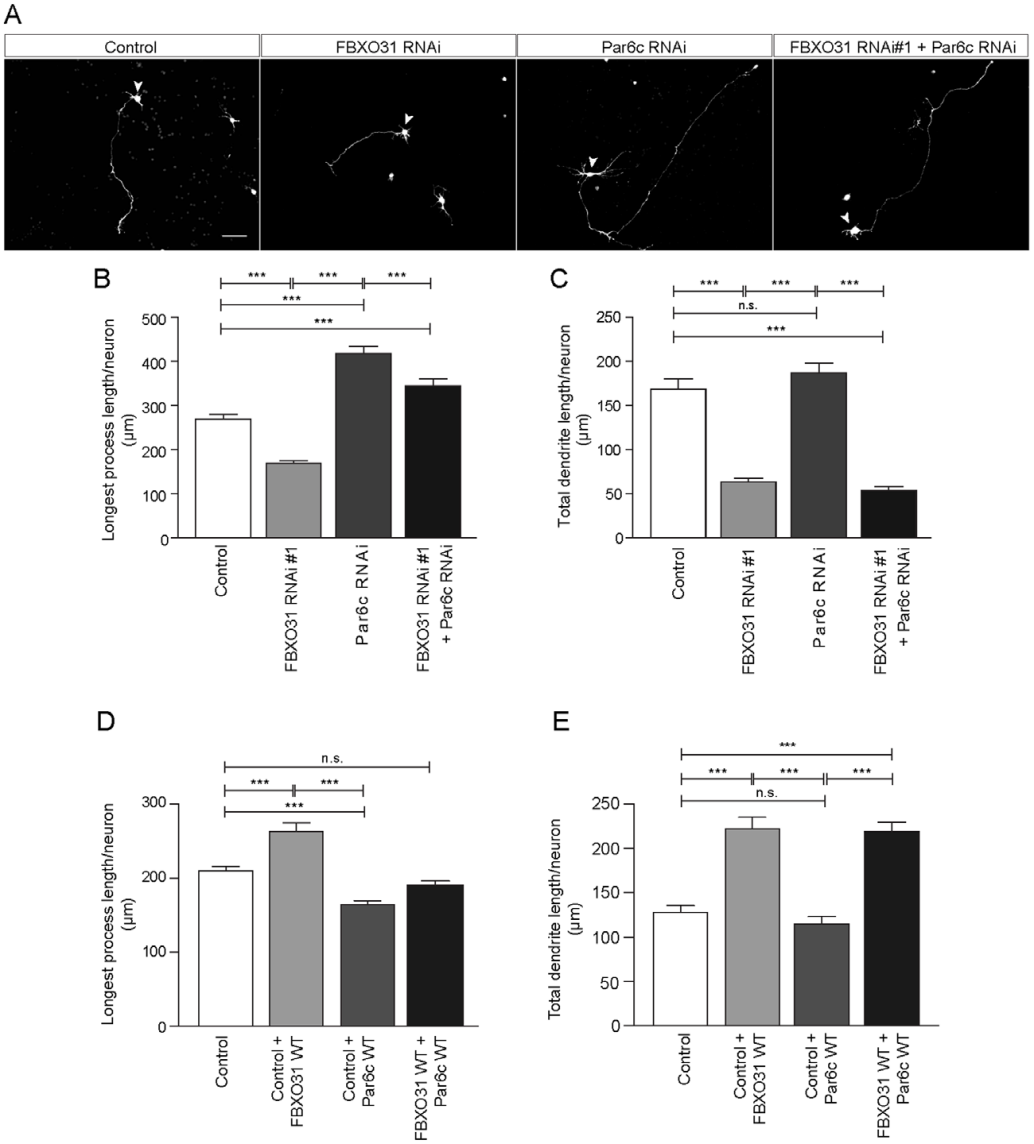
Par6c acts downstream of FBXO31-SCF in the control of axon growth. A. Representative images of cerebellar granule neurons transfected with control plasmid or FBXO31 RNAi #1 plasmid or Par6c RNAi plasmid or both FBXO31 RNAi #1 and Par6c RNAi plasmid together with GFP plasmid at DIV 0 and analyzed at DIV 4. Arrowheads indicate granule neuron cell bodies. Scale bar equals 50 µm. **B.** Quantification of longest process length of granule neurons shown in A (N = 3, n = 439, mean±SEM, one-way ANOVA, ***p<0.001). **C.** Quantification of total dendrite lengths of granule neurons shown in A (N = 3, n = 318, mean±SEM, one-way ANOVA, ***p<0.001, n.s. = not significant). **D.** Quantification of longest process length of granule neurons transfected with control plasmid or FBXO31 WT or Par6c WT or both FBXO31 WT and Par6c WT plasmid together with GFP plasmid at DIV 0 and analyzed at DIV 3. (N = 3, n = 387, mean±SEM, one-way ANOVA, ***p<0.001, n.s. = not significant). **E.** Quantification of total dendrite length of granule neurons transfected with control plasmid or FBXO31 WT or Par6c WT or both FBXO31 WT and Par6c WT plasmid together with GFP plasmid at DIV 0 and analyzed at DIV 3. (N = 3, n = 351, mean±SEM, one-way ANOVA, ***p<0.001, n.s. = not significant).

Since we identified Par6c as a target of FBXO31-SCF and a regulator of longest process growth, we reasoned that Par6c acts as a downstream component in the FBXO31-SCF pathway of neuronal morphogenesis. To establish such a cascade, we carried out epistasis analysis and transfected neurons with the FBXO31 RNAi or the Par6c RNAi plasmid or both and measured longest process length and dendritic lengths. While we found that FBXO31 RNAi reduces longest process and dendrite length and Par6c RNAi stimulates longest process growth, FBXO31/Par6c double knockdown results in enhanced growth longest processes and short dendrites. These results demonstrate that although the Par6c RNAi phenotype is dominant over FBXO31 RNAi phenotype regarding the longest process, the dendritic FBXO31 phenotype prevails (**Figure 5A–C**). In an analogous epistasis experiment, we overexpressed FBOX31 and Par6c individually or together and found that while FBXO31 increases and Par6c decreases longest process length, expression of Par6c and FBXO31 together results in reduced axonal length but has no effect on dendrites (**Figure 5D, 5E**). These data support the conclusion that Par6c acts downstream of FBXO31-SCF in longest process growth control, but not in dendrite growth.

### FBXO31-SCF Promotes Dendrite Growth and Migration in the Developing Cerebellum

**Figure 6:**
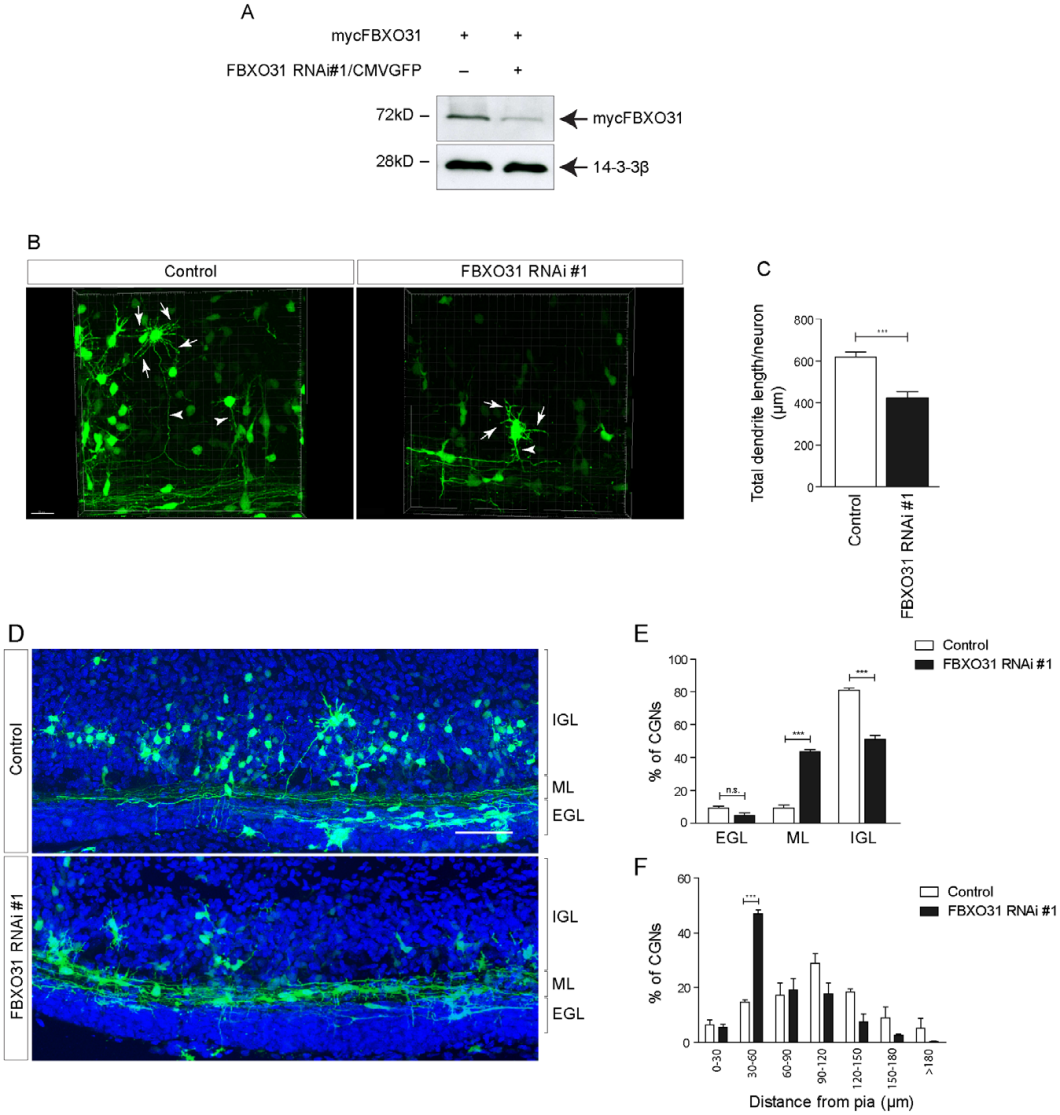
FBXO31 regulates dendrite growth and neuronal migration in the developing cerebellum. A. HEK 293T cell lysates transfected with mycFBXO31 along with control or bi-cistronic FBXO31 RNAi #1/CMV-GFP plasmids were probed with α-myc antibody. 14-3-3ß served as a loading control. **B.** Snapshots of 3D-reconstructed cerebellar granule neurons from rat pups electroporated with control plasmid or with FBXO31 RNAi #1/CMV-GFP bi-cistronic plasmid at P4 and analyzed at P9. Arrows indicate dendrites and arrowheads indicate axons of granule neurons. Scale bar equals 50 µm. **C.** Histogram showing dendrite length measurements for control or FBXO31 knockdown neurons. A total of 84 neurons were analyzed for dendrite length measurements (n = 3, mean±SEM, unpaired t-test, ***p<0.001). **D.** Coronal sections of rat pup cerebellum electroporated as described in Figure 4B. IGL = internal granular layer, ML = molecular layer, EGL = external granular layer. Scale bar equals 50 µm. **E.** Histogram showing percentage of migrated neurons in EGL, ML or IGL. A total of 3637 neurons were analyzed. (n = 3, mean±SEM, two-way ANOVA ***p<0.001, n.s. = not significant). **F.** Histogram showing distance of granule neuron cell bodies from the pial surface. A total of 681 neurons were analyzed. (n = 3, mean±SEM, two-way ANOVA ***p<0.001).

Finally, we explored the role of FBXO31 in the context of the developing cerebellum. We generated a bicistronic plasmid encoding the small hairpin RNA, we have previously used in cultured neurons, and a GFP cassette. We verified the efficient knockdown of FBXO31 by this bicistronic plasmid in heterologous cells (**Figure 6A**). We then electroporated the cerebella of P4 rat pups and isolated the tissue 5 days later. In coronal sections of P9 cerebella, we assessed dendrite growth of the GFP-positive neurons in the internal granular layer and found a reduction in total dendritic length in FBXO31 knockdown condition as compared to control (**Figure 6B, 6C**). Due to technical limitations, it is not feasible to measure axons as they fasciculate in the molecular layer into untraceable fibers. Strikingly, we also found that while more than 80% of transfected control neurons descend rapidly into the internal granular layer, nearly 50% of FBXO31 RNAi neurons fail to migrate and remain in the external granule layer/molecular layer (**Figure 6D, 6E** **).** We further quantified the migration defect and measured the distance of transfected neurons from the pial surface and found that the migration of FBXO31 knockdown neurons is markedly stalled (**Figure** **6F**). These results indicate that FBXO31 promotes dendritic morphogenesis and migration of granule neurons in the developing cerebellum.

## Discussion

In this study, we described that the novel centrosomal E3 ligase FBXO31-SCF regulates neuronal morphogenesis by targeting Par6c for proteasomal degradation. In addition, we found that FBXO31 is required for proper axonal identity and the efficient migration of neurons in the cerebellar cortex.

The E3 ligase FBXO31-SCF has previously been implicated in cell cycle regulation as it emerged as a tumor suppressor and regulator of DNA repair by degrading cyclin D1 [17], [18]. Its abundant expression in the nervous system has been reported [17], which is consistent with our data suggesting a relative enrichment of FBXO31 in neural tissue.

We identified FBXO31 as a regulator of axonal and dendritic growth. FBXO31-SCF follows the lead of the E3 ligase Cdh1-APC, which is a crucial cell cycle regulator with an unexpected role in axon growth regulation in neurons by targeting the substrates SnoN and Id2 for proteasomal degradation [33]–[35]. Like FBXO31-SCF, the E3 ligase FBXW7-SCF also operates in the cerebellum, where it controls cerebellar size, Purkinje cell number and axonal arborization [11]. In addition, other F-box proteins were identified to support the proper functioning of neurons including brain-specific FBXO45, which is required for formation of axon tracts and neuromuscular junctions in mammals, its C. elegans homologue FSN-1, which regulates presynaptic differentiation [13], [15], and lin-23 (FBXW1) that regulates the abundance of glutamate receptors [14]. Furthermore, the F-box protein FBXW8, which forms a Cullin-7 based E3 ligase, regulates the morphology of the Golgi complex and dendrites by degrading the Golgi protein Grasp65 [16]. These studies and our report support the notion of an elaborate interplay of F-box proteins in neurons.

The finding that F-box protein FBXO31 is a centrosomal protein in neurons is in agreement with FBXO31 being a regulator of neuronal morphogenesis, axon specification and migration. The centrosome has been previously identified as a cornerstone of neuronal polarity allowing the prediction of the positioning of the future axon [36]. Also, the centrosome’s function as microtubule organizing center (MTOC) marks not only the site of axonal outgrowth but is intimately connected with the coordination of neuronal migration [37]–[39]. FBXO31-SCF joins a small number of E3 ligases, which are positioned at the centrosome including dendrite-regulating Cdc20-APC and the Parkinson’s disease protein Parkin [40]–[42], supporting an essential role for centrosomal E3 ubiquitin ligases in neuronal development and disease [43], [44].

FBXO31’s centrosomal position led us to discover the polarity protein Par6c as an interactor and novel substrate. Par6c has previously been described as a component of the centrosome, which recruits other centrosomal proteins [24]. A key function of Par6, which forms the Par complex together with aPKC and Par3b, is its role in neuronal polarity [27], [28], [45], [46]. Also, the Par6 complex has been implicated in stimulation-induced local control of axon growth [47]. Yet another study proposed that Par6 acts on its own as an effector of TGF beta-induced axon initiation and growth via type II TGF-beta receptor [48]. Our finding that Par6c inhibits axon growth but has no effect on dendrites ascribes a specific axon growth-suppressing role to Par6c. These results are supported by previous work that revealed a Par6-mediated inhibition of axon growth [49]. We believe that Par6c acts in an alternative complex in axon growth control at the centrosome since we did not find an interaction of FBXO31 with Par3b (neither in the presence nor absence of Par6c; data not shown). Our results also underscore a dual role for Par6c in neuronal polarity and in axon growth control. The latter may require other Par6c interactors than Par3b. A study by Zhang and Macara revealed that dendritic spine development is yet another aspect in neurons that is regulated by Par6 but not Par3b [32].

Apart from FBXO31-SCF, Par6c is degraded by the E3 ligase Smurf1 in axon initiation [50]. Here, Smurf1 promotes Par6c degradation to initiate axons but then switches to RhoA degradation for axon elongation as a consequence of trophic factor-induced phosphorylation of Smurf1. In contrast to FBXO31, Smurf1 has not been described as a centrosomal E3 ligase. FBXO31 together with Smurf1 and TGFß signaling is likely to orchestrate axonal initiation and growth by keeping the common substrate Par6 in check at different subcellular localizations and in response to various extrinsic cues.

The finding that FBXO31 controls dendritic growth but not via Par6c also prompted us to conclude that FBXO31-SCF is likely to target another protein for degradation in the control of dendrite growth. Our *in vivo* analyses underscored FBXO31’s importance in proper dendrite development in the cerebellum. Furthermore, our results implicate that FBXO31-SCF promotes neuronal migration in the cerebellum, which is consistent with the finding of Solecki and colleagues who reported abated migration of granule neurons in Par6 gain-of-function analyses in organotypic cerebellar slices and hence identified Par6 as a negative regulator of neuronal migration in the cerebellum [49]. It is very well conceivable that the FBXO31-SCF/Par6 pathway regulates in addition to axon growth, migration of granule neurons in the cerebellum.

Our results expand our knowledge on F-box proteins in the control of neuronal morphology by giving a first glimpse into FBXO31-regulated events in developing neurons and introducing the E3 ligase FBXO31-SCF as a potential key regulator of neuronal development. Our findings also set the stage for future research, which should identify other substrates of this ligase to establish an FBXO31-SCF-controlled signaling network in neurons. Interestingly, a recent report revealed a drop in FBXO31 levels in schizophrenic patients with short term illness, implicating FBXO31 in schizophrenia [51]. Hence, it will be highly informative to determine how systemic or conditional deletions of FBXO31 affect brain development and if defects and phenotypes resulting thereof are reminiscent of developmental brain disorders including schizophrenia.

## Supporting Information

Figure S1 **FBXO31 expression and localization. A. to C.** quantitative PCR analysis of FBXO31 expression in various tissues of postnatal day (P) 6 rat pup (A), P12 rat pup (B) and adult rat (C). Data was normalized to β-actin and values indicated are relative to cortex for each group. **D.** Hippocampal neurons cultured from E18 rat embryos were immunostained with α-FBXO31 antibody with or without pre-incubation with recombinant FBXO31 protein recognized by the FBXO31 antibody. Arrows indicate centrosome. Scale bar equals 10 µm.
(TIF)

Figure S2 **FBXO31 associates with Cullin1 and Skp1 through its F-box domain.** HEK 293T cells were co-transfected with mycSkp1 and GFP-FBXO31 WT or ΔF plasmids together with respective control vectors. Cell lysates were subjected to immunoprecipitation with α-GFP antibody and immunoblotted with α-Cul1 and α-myc antibodies.
(TIF)

Figure S3 **The F-box domain of FBXO31 is not essential for its centrosomal localization. A.** Schematic of FBXO31 deletion mutants and their respective sub-cellular localization. **B.** HEK 293T cells were transfected with indicated GFP-FBXO31 deletion mutant constructs together with Flag-DISC1 plasmid and immunostained with anti-GFP and anti-Flag antibodies. The cells were counterstained with the DNA dye bisbenzimide Hoechst 33258. Scale bar equals 10 µm.
(TIF)

Figure S4 **FBXO31 promotes axon and dendrite growth in cerebellar granule neurons. A.** HEK 293T cell lysates transfected with mycFBXO31 along with control or FBXO31 RNAi #1, #2 or #3 plasmids were probed with α-myc antibody. 14-3-3ß served as a loading control. Note that FBXO31 RNAi #2 is non-functional. **B.** Representative images of granule neurons transfected with empty control vectors, FBXO31 RNAi #1, #2 or #3 plasmid at DIV 0 and analyzed at DIV 4. Arrowheads indicate granule neuron cell bodies. Scale bar equals 50 µm. **C. and D.** Quantification of longest process lengths (C) (N = 3, n = 539, mean±SEM, one-way ANOVA, ***p<0.001) and total dendrite length (D) (N = 3, n = 526, mean±SEM, one-way ANOVA, ***p<0.001) of cerebellar granule neurons transfected with control vector or FBXO31 RNAi#1, #2 or #3 plasmids together with GFP plasmid at DIV 0 and analyzed at DIV 4. **E.** Quantification of percentage of apoptotic granule neurons transfected with control, FBXO31 RNAi #1, #2 or #3 plasmids together with ß-galactosidase plasmid at DIV 2 and analyzed at DIV 6 (N = 3, n = 1585, mean±SEM, one-way ANOVA, ***p<0.001, **p<0.01, *p<0.05, n.s. = not significant).
(TIF)

Figure S5 **Biochemical characterization of the FBXO31-Par6c interaction. A.** Lysates of HEK 293T cells transfected with plasmids encoding GFP-FBXO31 and Par6c deletion mutants were subjected to immunoprecipitation with α-myc antibody and immunoblotted with anti-GFP antibody. **B. and C.** HEK 293T cells (B) and granule neurons (C) were transfected with mycPar6c plasmid along with FBXO31 WT plasmids or respective control vectors as indicated. Cell lysates were immunoblotted with α-myc and α-Flag antibodies. 14-3-3ß served as a loading control. **D.** Lysates of granule neurons transfected with control vector or FBXO31 RNAi #1 plasmid were subjected to centrosomal purification. Shown here is the immunoblotting analysis of non-centrosomal protein-containing supernatant of the first ultracentrifugation step revealing the presence of the cytoplasmic protein 14-3-3ß. **E.** HEK 293T cells were co-transfected with mycPar6c and GFP-FBXO31 WT or ΔF plasmids along with respective control vectors. Cell lysates were denatured and subjected to immunoprecipitation with anti-myc antibody and immunoblotted with K48 linkage-specific anti-ubiquitin antibody.
(TIF)

Figure S6 **The FBXO31-SCF target Cyclin D1 does not influence axon and dendrite growth in cerebellar granule neurons. A.** Representative images of cerebellar granule neurons transfected with empty control vector or GFP-Cyclin D1 plasmid at DIV 0 and analyzed at DIV 3. Arrowheads indicate granule neuron cell bodies. Scale bar equals 50 µm. **B.** Quantification of longest process lengths of granule neurons shown in A (N = 3, n = 197, mean±SEM, unpaired t-test, n.s. = not significant). **C.** Quantification of total dendrite lengths of granule neurons shown in A (N = 3, n = 194, mean±SEM, unpaired t-test, n.s. = not significant). **D.** Quantification of percentage of non-polarized granule neurons shown in A. (N = 3, n = 200, mean±SEM, unpaired t-test, n.s. = not significant).
(TIF)

Methods S1 **Quantitative RT-PCR.** cDNA synthesized from RNA isolated from various tissues (cortex, hippocampus, cerebellum, olfactory bulb, liver, lung, heart, spleen and kidney) of P4, P12 and 4 months old adult rat, was used for quantitative PCR (Roche light cycler). The primers used for *FBXO31* gene were: sense 5′ CCACTGTTTTAGAATCCATCTGATGGA 3′ and anti-sense 5′ ACTTGGTGGAGAACTCGTCCC 3′ while the primers used for *β-actin* were: sense 5′ CTTCCTCCCTGGAGAAGAGC 3′ and antisense 5′ ATGCCACAGGATTCCATACC 3′. The *FBXO31* levels were normalized to *β-actin* and represented relative to the cortex values for each age group.
(DOCX)

Methods S2 **Cell-based ubiquitination assay.** Transfected HEK 293T cells were lysed in RIPA buffer (50 mM Tris-HCl pH 8.0, 150 mM NaCl, 1% NP40, 0.5% sodium deoxycholate, 0.1% SDS and 5 mM EDTA) supplemented with fresh protease inhibitors (1 µg/mL pepstatin, 3 µg/mL aprotinin and 1 µg/mLleupeptin) and 10 mM NEM. 1 mg of total protein was boiled in 1% SDS for 5 minutes at 95 °C, diluted 10x in lysis buffer (50 mM HEPES pH 7.5, 150 mM NaCl, 10% glycerol, 1.5 mM MgCl_2_, 1% Triton X-100) to dilute the SDS and immunoprecipitated with anti-myc antibody for 2 hours at 4 °C. 50 µL of Protein A sepharose beads were added to the lysates and incubated for 1 hour at 4 °C. The samples were washed twice with lysis buffer, twice with HNTG buffer (20 mM HEPES pH 7.5, 150 mM NaCl, 10% glycerol and 0.1% Triton X-100), once with PBS and boiled with SDS sample buffer.
(DOCX)
