# Supplementary figures and images for: The Centrosomal E3 Ubiquitin Ligase FBXO31-SCF Regulates Neuronal Morphogenesis and Migration
Source: PLoS One. 2013 Feb 28;8(2):e57530. doi: 10.1371/journal.pone.0057530 (PMC3585373; doi:10.1371/journal.pone.0057530)

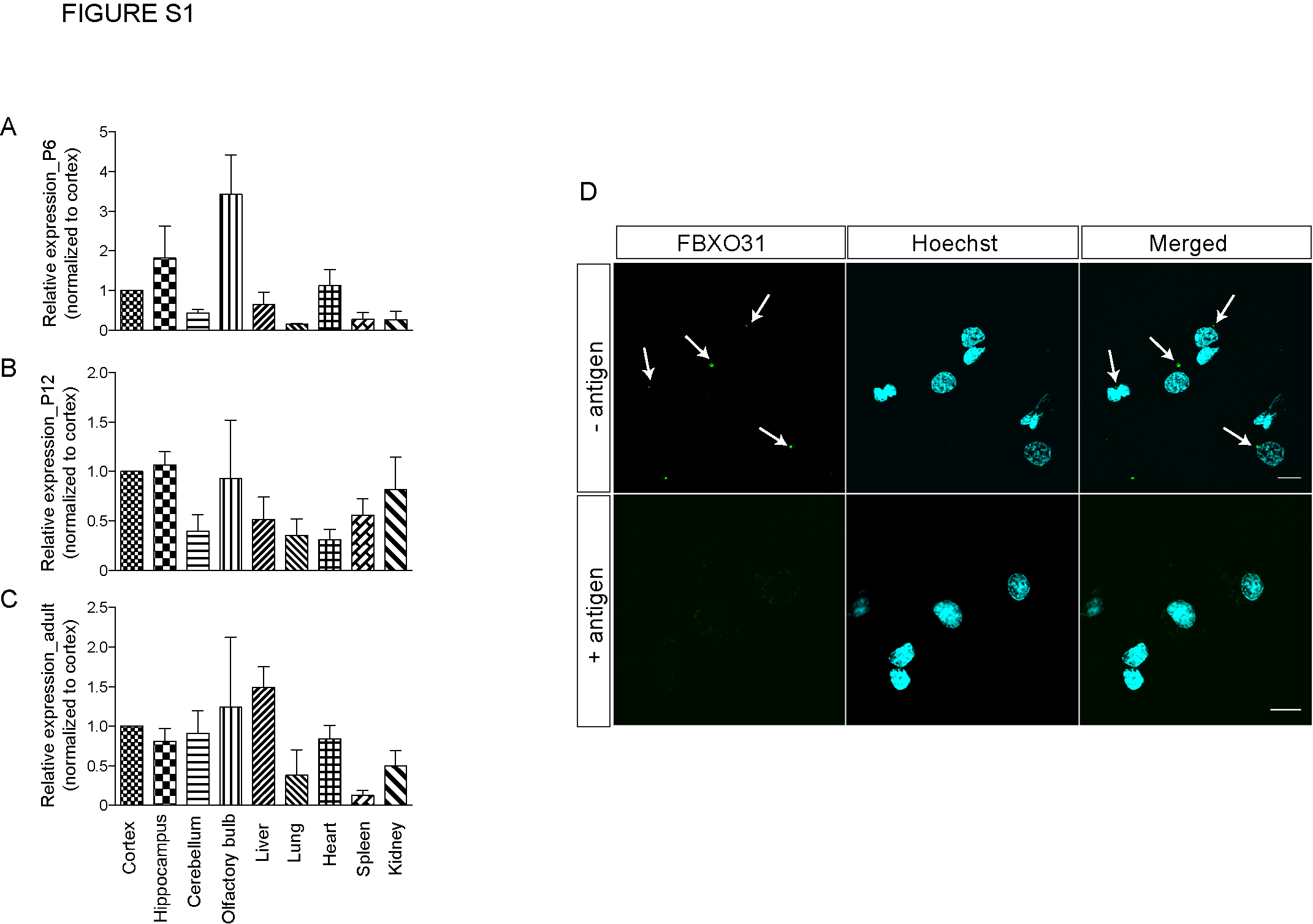

Supplement: Figure S1 — FBXO31 expression and localization. A. to C. quantitative PCR analysis of FBXO31 expression in various tissues of postnatal day (P) 6 rat pup (A), P12 rat pup (B) and adult rat (C). Data was normalized to β-actin and values indicated are relative to cortex for each group. D. Hippocampal neurons cultured from E18 rat embryos were immunostained with α-FBXO31 antibody with or without pre-incubation with recombinant FBXO31 protein recognized by the FBXO31 antibody. Arrows indicate centrosome. Scale bar equals 10 µm. (TIF) [file pone.0057530.s001.tif]

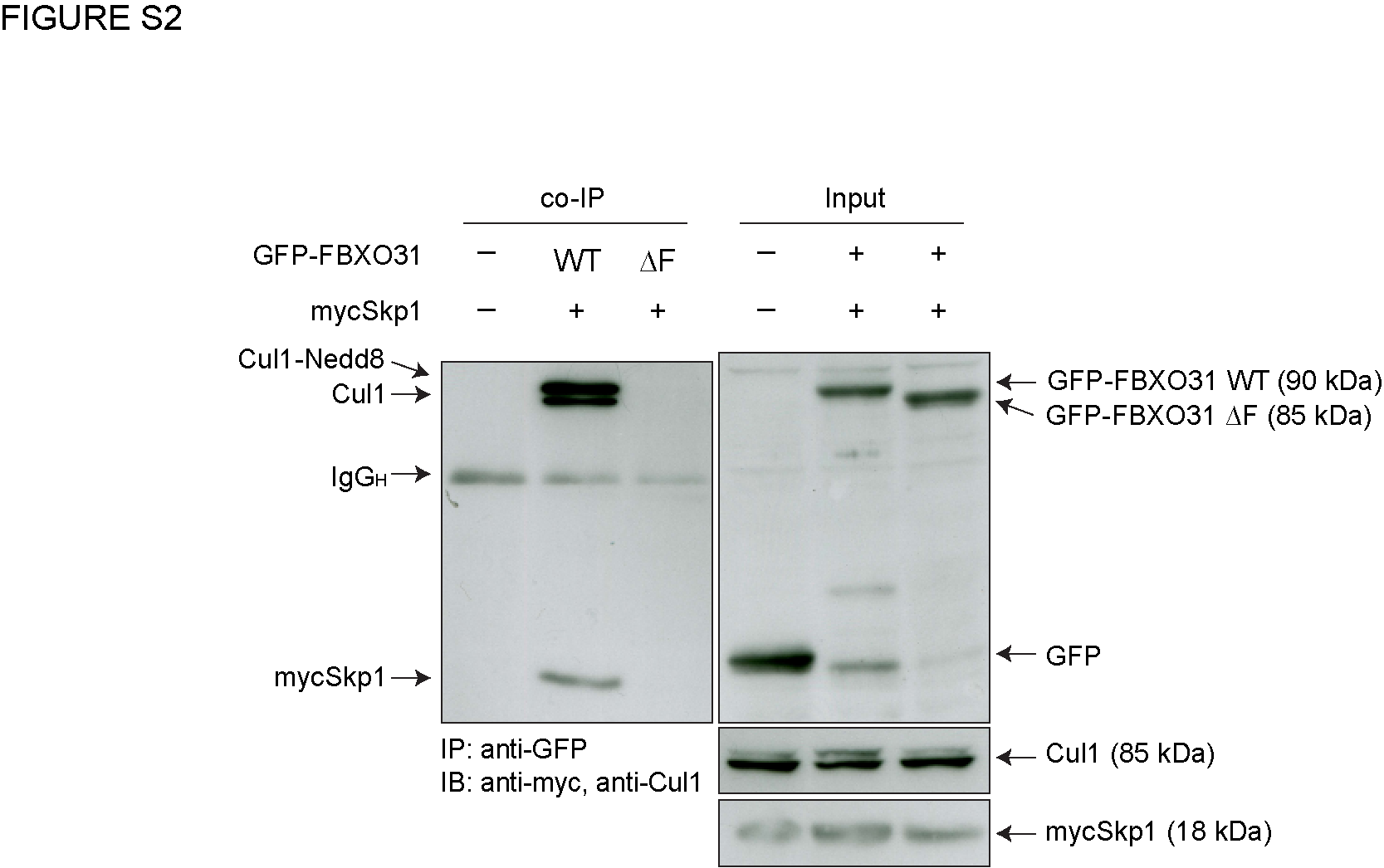

Supplement: Figure S2 — FBXO31 associates with Cullin1 and Skp1 through its F-box domain. HEK 293T cells were co-transfected with mycSkp1 and GFP-FBXO31 WT or ΔF plasmids together with respective control vectors. Cell lysates were subjected to immunoprecipitation with α-GFP antibody and immunoblotted with α-Cul1 and α-myc antibodies. (TIF) [file pone.0057530.s002.tif]

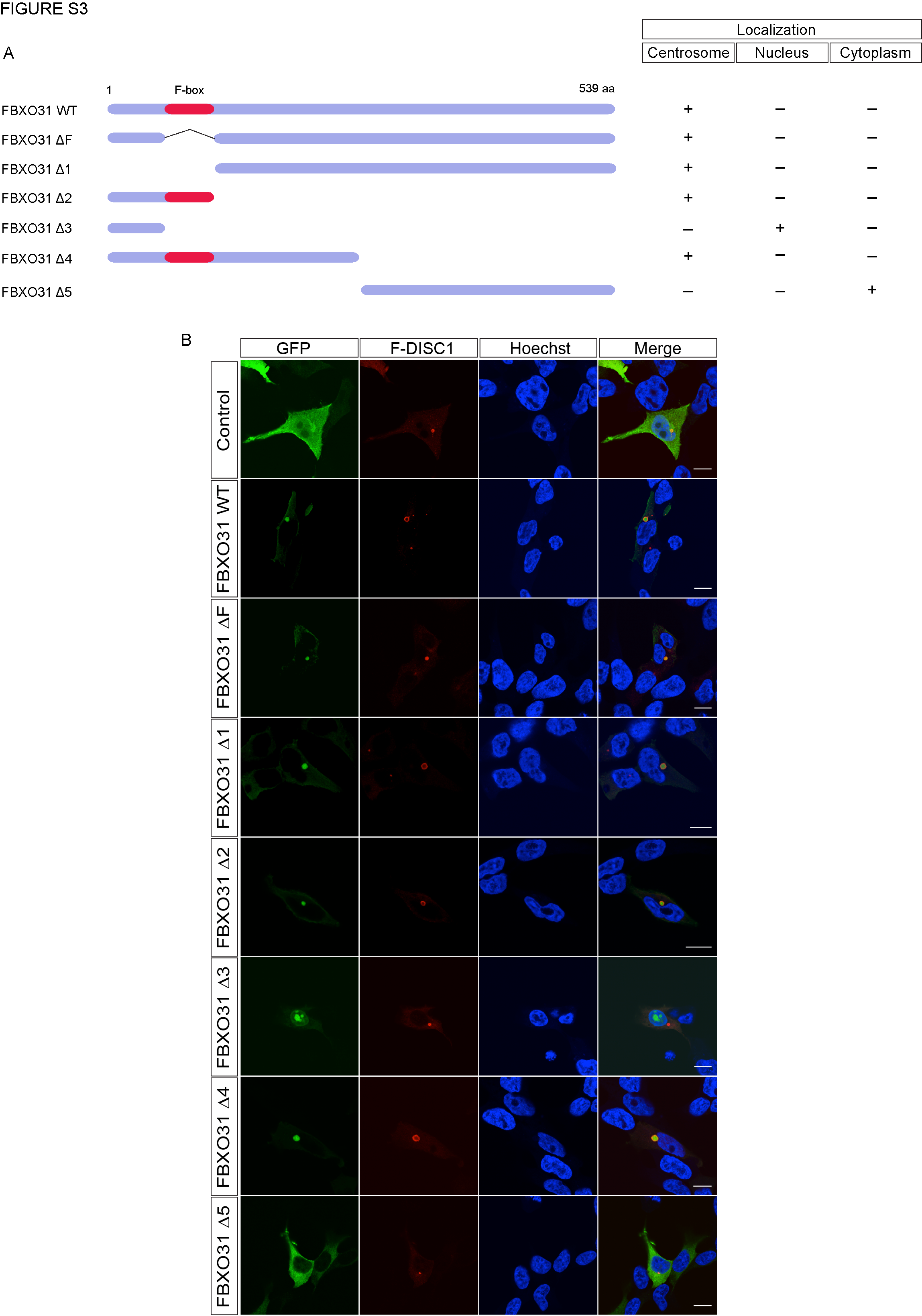

Supplement: Figure S3 — The F-box domain of FBXO31 is not essential for its centrosomal localization. A. Schematic of FBXO31 deletion mutants and their respective sub-cellular localization. B. HEK 293T cells were transfected with indicated GFP-FBXO31 deletion mutant constructs together with Flag-DISC1 plasmid and immunostained with anti-GFP and anti-Flag antibodies. The cells were counterstained with the DNA dye bisbenzimide Hoechst 33258. Scale bar equals 10 µm. (TIF) [file pone.0057530.s003.tif]

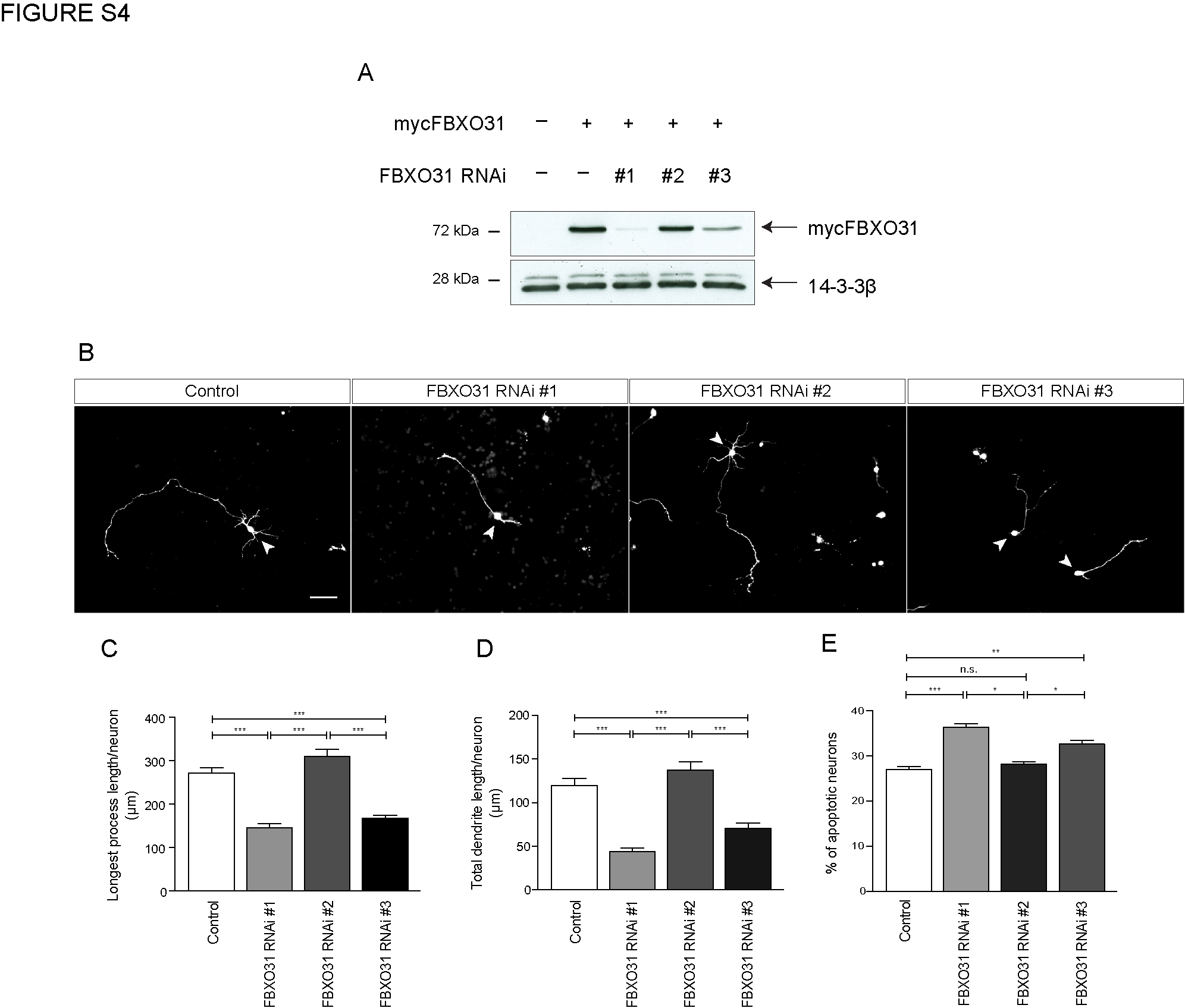

Supplement: Figure S4 — FBXO31 promotes axon and dendrite growth in cerebellar granule neurons. A. HEK 293T cell lysates transfected with mycFBXO31 along with control or FBXO31 RNAi #1, #2 or #3 plasmids were probed with α-myc antibody. 14-3-3ß served as a loading control. Note that FBXO31 RNAi #2 is non-functional. B. Representative images of granule neurons transfected with empty control vectors, FBXO31 RNAi #1, #2 or #3 plasmid at DIV 0 and analyzed at DIV 4. Arrowheads indicate granule neuron cell bodies. Scale bar equals 50 µm. C. and D. Quantification of longest process lengths (C) (N = 3, n = 539, mean±SEM, one-way ANOVA, ***p<0.001) and total dendrite length (D) (N = 3, n = 526, mean±SEM, one-way ANOVA, ***p<0.001) of cerebellar granule neurons transfected with control vector or FBXO31 RNAi#1, #2 or #3 plasmids together with GFP plasmid at DIV 0 and analyzed at DIV 4. E. Quantification of percentage of apoptotic granule neurons transfected with control, FBXO31 RNAi #1, #2 or #3 plasmids together with ß-galactosidase plasmid at DIV 2 and analyzed at DIV 6 (N = 3, n = 1585, mean±SEM, one-way ANOVA, ***p<0.001, **p<0.01, *p<0.05, n.s. = not significant). (TIF) [file pone.0057530.s004.tif]

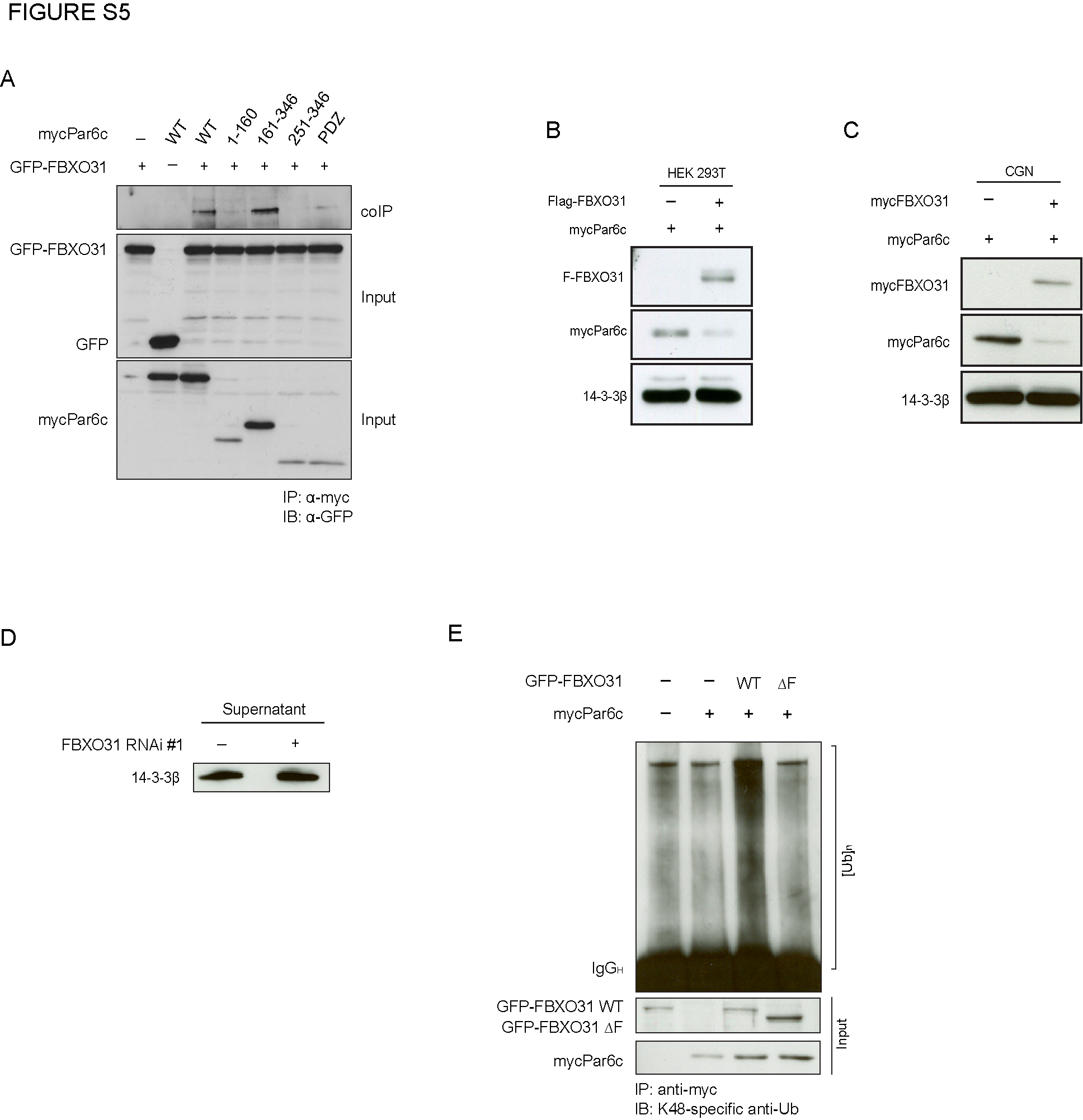

Supplement: Figure S5 — Biochemical characterization of the FBXO31-Par6c interaction. A. Lysates of HEK 293T cells transfected with plasmids encoding GFP-FBXO31 and Par6c deletion mutants were subjected to immunoprecipitation with α-myc antibody and immunoblotted with anti-GFP antibody. B. and C. HEK 293T cells (B) and granule neurons (C) were transfected with mycPar6c plasmid along with FBXO31 WT plasmids or respective control vectors as indicated. Cell lysates were immunoblotted with α-myc and α-Flag antibodies. 14-3-3ß served as a loading control. D. Lysates of granule neurons transfected with control vector or FBXO31 RNAi #1 plasmid were subjected to centrosomal purification. Shown here is the immunoblotting analysis of non-centrosomal protein-containing supernatant of the first ultracentrifugation step revealing the presence of the cytoplasmic protein 14-3-3ß. E. HEK 293T cells were co-transfected with mycPar6c and GFP-FBXO31 WT or ΔF plasmids along with respective control vectors. Cell lysates were denatured and subjected to immunoprecipitation with anti-myc antibody and immunoblotted with K48 linkage-specific anti-ubiquitin antibody. (TIF) [file pone.0057530.s005.tif]

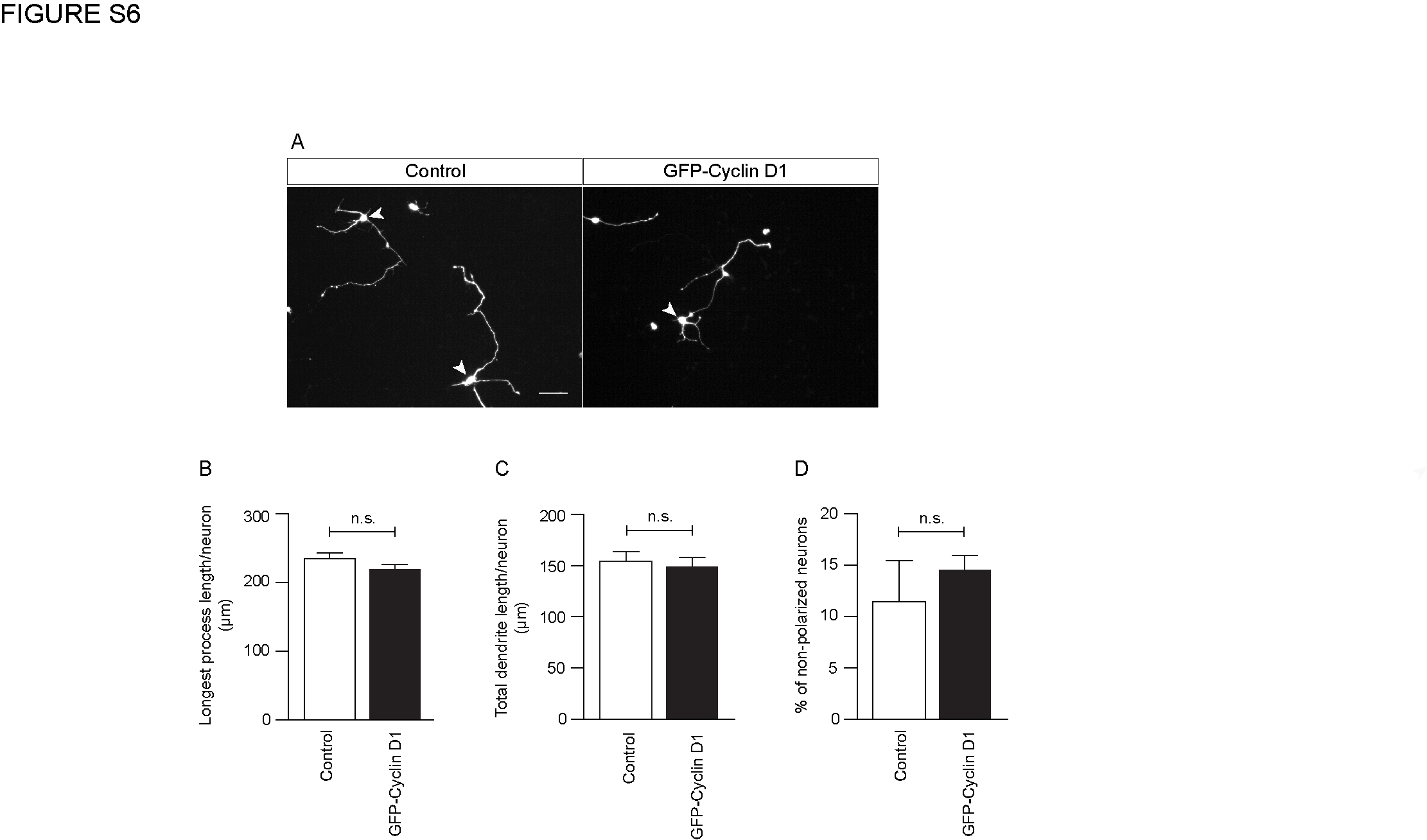

Supplement: Figure S6 — The FBXO31-SCF target Cyclin D1 does not influence axon and dendrite growth in cerebellar granule neurons. A. Representative images of cerebellar granule neurons transfected with empty control vector or GFP-Cyclin D1 plasmid at DIV 0 and analyzed at DIV 3. Arrowheads indicate granule neuron cell bodies. Scale bar equals 50 µm. B. Quantification of longest process lengths of granule neurons shown in A (N = 3, n = 197, mean±SEM, unpaired t-test, n.s. = not significant). C. Quantification of total dendrite lengths of granule neurons shown in A (N = 3, n = 194, mean±SEM, unpaired t-test, n.s. = not significant). D. Quantification of percentage of non-polarized granule neurons shown in A. (N = 3, n = 200, mean±SEM, unpaired t-test, n.s. = not significant). (TIF) [file pone.0057530.s006.tif]
