# Supplementary material for: The Centrosomal E3 Ubiquitin Ligase FBXO31-SCF Regulates Neuronal Morphogenesis and Migration
Source: PLoS One. 2013 Feb 28;8(2):e57530. doi: 10.1371/journal.pone.0057530 (PMC3585373; doi:10.1371/journal.pone.0057530)
Supplement: Methods S1 — Quantitative RT-PCR. cDNA synthesized from RNA isolated from various tissues (cortex, hippocampus, cerebellum, olfactory bulb, liver, lung, heart, spleen and kidney) of P4, P12 and 4 months old adult rat, was used for quantitative PCR (Roche light cycler). The primers used for FBXO31 gene were: sense 5′ CCACTGTTTTAGAATCCATCTGATGGA 3′ and anti-sense 5′ ACTTGGTGGAGAACTCGTCCC 3′ while the primers used for β-actin were: sense 5′ CTTCCTCCCTGGAGAAGAGC 3′ and antisense 5′ ATGCCACAGGATTCCATACC 3′. The FBXO31 levels were normalized to β-actin and represented relative to the cortex values for each age group. (DOCX) [file pone.0057530.s007.docx]

**METHODS S1**

*Quantitative RT-PCR*

cDNA synthesized from RNA isolated from various tissues (cortex, hippocampus, cerebellum, olfactory bulb, liver, lung, heart, spleen and kidney) of P4, P12 and 4 months old adult rat, was used for quantitative PCR (Roche light cycler). The primers used for *FBXO31* gene were: sense 5’ CCACTGTTTTAGAATCCATCTGATGGA 3’ and anti-sense 5’ ACTTGGTGGAGAACTCGTCCC 3’ while the primers used for *β-actin* were: sense 5’ CTTCCTCCCTGGAGAAGAGC 3’ and antisense 5’ ATGCCACAGGATTCCATACC 3’. The *FBXO31* levels were normalized to *β-actin* and represented relative to the cortex values for each age group.
