# Supplementary material for: The Centrosomal E3 Ubiquitin Ligase FBXO31-SCF Regulates Neuronal Morphogenesis and Migration
Source: PLoS One. 2013 Feb 28;8(2):e57530. doi: 10.1371/journal.pone.0057530 (PMC3585373; doi:10.1371/journal.pone.0057530)
Supplement: Methods S2 — Cell-based ubiquitination assay. Transfected HEK 293T cells were lysed in RIPA buffer (50 mM Tris-HCl pH 8.0, 150 mM NaCl, 1% NP40, 0.5% sodium deoxycholate, 0.1% SDS and 5 mM EDTA) supplemented with fresh protease inhibitors (1 µg/mL pepstatin, 3 µg/mL aprotinin and 1 µg/mLleupeptin) and 10 mM NEM. 1 mg of total protein was boiled in 1% SDS for 5 minutes at 95 °C, diluted 10x in lysis buffer (50 mM HEPES pH 7.5, 150 mM NaCl, 10% glycerol, 1.5 mM MgCl2, 1% Triton X-100) to dilute the SDS and immunoprecipitated with anti-myc antibody for 2 hours at 4 °C. 50 µL of Protein A sepharose beads were added to the lysates and incubated for 1 hour at 4 °C. The samples were washed twice with lysis buffer, twice with HNTG buffer (20 mM HEPES pH 7.5, 150 mM NaCl, 10% glycerol and 0.1% Triton X-100), once with PBS and boiled with SDS sample buffer. (DOCX) [file pone.0057530.s008.docx]

**METHODS S2**

*Cell-based ubiquitination assay*

Transfected HEK 293T cells were lysed in RIPA buffer (50 mM Tris-HCl pH 8.0, 150 mM NaCl, 1% NP40, 0.5% sodium deoxycholate, 0.1% SDS and 5 mM EDTA) supplemented with fresh protease inhibitors (1 µg/mL pepstatin, 3 µg/mL aprotinin and 1 µg/mLleupeptin) and 10 mM NEM. 1 mg of total protein was boiled in 1% SDS for 5 minutes at 95 ºC, diluted 10x in lysis buffer (50 mM HEPES pH 7.5, 150 mM NaCl, 10% glycerol, 1.5 mM MgCl_2_, 1% Triton X-100) to dilute the SDS and immunoprecipitated with anti-myc antibody for 2 hours at 4 ºC. 50 µL of Protein A sepharose beads were added to the lysates and incubated for 1 hour at 4 ºC. The samples were washed twice with lysis buffer, twice with HNTG buffer (20 mM HEPES pH 7.5, 150 mM NaCl, 10% glycerol and 0.1% Triton X-100), once with PBS and boiled with SDS sample buffer.
